# Reviews

**Published:** 1884-02

**Authors:** 


### Reviews.

THE PATHOLOGY, DIAGNOSIS, AND TREATMENT OF DISEASES OF WOMEN. By GRAILY HEWITT, M.D. London, F.R.C.P., etc., etc. Edited with Notes and Additions by Harry Marion Sims, M.D., New York. A New American from the Fourth Revised and Enlarged London Edition. With 236 Illustrations Bermingham & Co., New York: 1883. Two Vols., each $2.25.

Dr. Hewitt needs no introduction to American readers. His work on the Diseases of Women has been conspicuously before the medical public for twenty years. Its first edition appeared in October, 1863. It has been enlarged with each reissue, till its present appearance is in two volumes of over 500 pages each. Every gynæcologic subject is treated in Dr. Hewitt's charming and forceful manner. To ripe scholarship, he has added an extensive and varied experience, and it is this happy combination that makes Professor Hewitt an authority in gynæcology. The chapters on Cancer; the Hystero-Neuroses; the Abnormalities of Menstruation, and the Diseases of the Ovaries, are especially interesting and instructive. Fourteen years ago, the writer heard Professor Hewitt lecture on uterine flexions and displacements, and saw him adjust many pessaries, and was greatly impressed with the extreme readiness with which he selected and adjusted the latter. Upon this branch of the subject we may turn to the fullest chapters in the work, with the assurance of reading the most complete exposition of it in any language. Professor Hewitt is the especial advocate of the mechanical cause of uterine disorders. This belief is defended and advocated most elaborately.

The work is well issued, and will be an advantage and an ornament to any library.

? QUIZ COMPENDS ? No. 6. A Compend on Materia Medica and Therapeutics, With Especial Reference to the Physiological Action of Drugs. For the Use of Medical, Dental and Pharmaceutical Students. Based on the Sixth Decennial Revision of the U. S. Pharmacopœia, and Including many Unofficial Remedies. By SAMUEL O. POTTER, M.A., M.D., Acting Assistant Surgeon U. S. Army. Philadelphia: P. Blakiston, Son & Co. 1883. Pp. 141.

A most excellent work for students. Preceptors cannot put into the hands of their students any book that will so well drill them in the subjects of materia medica and therapeutics, as will this small volume. Brevity of statement is one of its principal features. At the same time, the essentials of the subjects have been kept in view. Each drug is methodieally handled. First, its materia medica is given; then its official preparations. Afterwards follow its physiological action and therapeutics. Altogether, it is the completest "quiz compend" extant.

SEPULTURE: Its History, Methods and Sanitary Requisites. By STEPHEN WICKES, A.M., M.D., Author of "History of Medicine aad Medical Men of New Jersey," etc. Philadelphia: P. Blakiston, Son & Co. 1884. Pp. 156.

Its table of contents enumerates the following subjects: History of Sepulture; Ancient Customs and Methods; Sepulchres; Interments Among the Greeks; Customs Among the Romans; Persian Burial; North American Indian Burial; Early Christian Burial; Animal Putrescence; Malignant Disease from One Corpse; Saturated Soil of a Graveyard Disturbed; Intramural Interment in the United States; Yellow Fever; Asiatic Cholera; Pestilence; Rural Cemeteries; Coffins for the Dead of Country Graveyards.

The author has evidently spent a vast deal of time in collating the facts that he incorporates in this volume. It is one of the outgrowths of the agitation of the subject of cremation, and is assuredly a most interesting and instructive work.

